# Exploring the Role of Infodemics in People’s Incompliance with Preventive Measures during the COVID-19 in Conflict Settings (Mixed Method Study)

**DOI:** 10.3390/healthcare11070952

**Published:** 2023-03-26

**Authors:** Ahmed Asa’ad Al-Aghbari, Ola El Hajj Hassan, Maureen Dar Iang, Albrecht Jahn, Olaf Horstick, Fekri Dureab

**Affiliations:** 1 Heidelberg Institute of Global Health, Heidelberg University Hospital, 69120 Heidelberg, Germany; ahmedalaghbari24@gmail.com (A.A.A.-A.);; 2 Institute of Research in International Assistance, Akkon-Hochschule für Humanwissenschaften, 12099 Berlin, Germany

**Keywords:** Infodemic management, health misinformation, information source, information access, health crises, incompliance, restriction on movement, COVID-19 preventive measures, social behaviors

## Abstract

The evolving availability of health information on social media, regardless of its credibility, raises several questions about its impact on our health decisions and social behaviors, especially during health crises and in conflict settings where compliance with preventive measures and health guidelines is already a challenge due to socioeconomic factors. For these reasons, we assessed compliance with preventive measures and investigated the role of infodemic in people’s non-compliance with COVID-19 containment measures in Yemen. To this purpose and to triangulate our data collection, we executed a mixed method approach in which raw aggregated data were taken and analyzed from multiple sources (COVID-19 Government Response Tracker and Google COVID-19 Community Mobility Reports), then complemented and verified with In-depth interviews. Our results showed that the population in Yemen had relatively complied with the governmental containment measures at the beginning of the pandemic. However, containment measures were not supported by daily COVID-19 reports due to low transparency, which, together with misinformation and lack of access to reliable sources, has caused the population not to believe in COVID-19 and even practice social pressure on those who showed some compliance with the WHO guidelines. Those results indicate the importance of adopting an infodemic management approach in response to future outbreaks, particularly in conflict settings.

## 1. Introduction

Ten years ago, for better or worse, social media evolved as part of our everyday lives and radically transformed how we live and communicate–both in our private and professional lives [1]. Today there are over 4.74 billion social media users worldwide, equating to 59.3 percent of the total global population [2]; where everyone is active on one social media platform or another, teenagers on TikTok, influencers and businesses on Instagram and Facebook, or professionals on LinkedIn–when looking for growing connections in any field, social media is the first choice that comes to mind [3]. Thus, using social media intelligently can bring remarkable success and benefits.

However, social media and instant messaging became potent sources of all kinds of misinformation in times of emergencies and health crises, as shown in studies focusing on Weibo in China [4] and Twitter in West Africa after the 2014–2015 Ebola outbreak [5]. This has revised the way organizations communicate with their stakeholders as well as provided new opportunities for stakeholders to engage in an immediate and direct dialogue both with organizations and each other [6].

The WHO acknowledges that for the coronavirus disease 2019 (COVID-19) pandemic, there is a tremendous amount of misinformation that makes it hard for people to find trustworthy sources and reliable guidance when needed [7]. A total lockdown of cities affects the productive and informal economic sector, conceivably leading to hunger and much-extended poverty, which, together with other factors like the spread of false information (Infodemic), caused the community not to comply with the governmental preventive measures [8,9]. The WHO defines an infodemic as “an acute outpouring of information, including potentially misleading or inaccurate information that, in a digital, hyper-connected society such as the present one, is likely bound to accompany every epidemic or acute health crisis.” [10] Dr. Tedros Adhanom Ghebreyesus, the Director of the World Health Organization (WHO), during the Munich Security Conference On 15 February 2020, articulated that the fight against the COVID-19 pandemic was accompanied by combat against an “infodemic,” [11]. 

This concern is not new: others have transpired during other health emergencies, but never one of the current magnitudes resulting from the risen use of digital platforms and Apps. In the era of digital interdependence, this phenomenon is amplified by increased access to mobile devices, internet access, and social networks, which are circulating it like a virus, further and faster than ever before [11]. The COVID-19 infodemic has formed widespread worry, anxiety, confusion, and suffering in the general public, significantly among persons who lack proper digital literacy and people with mental problems [12]. Uncertainty is a crucial source of chaos and is more stressful than knowing decisive negative consequences [13]. Anxiety is induced by panic-provoking media articles and alarming social media messages. During the 2020–2022 period, the COVID-19 outbreak has dominated the news, delivering a bleak, pessimistic overview of the world and life. In a vulnerable population like our population in Yemen, the denial of COVID-19 and the incompliance with preventive measures can be triggered by anxiety, anguish, and uncertainty [13]. 

After COVID-19 had reached the Arabic peninsula and evolved into a pandemic, Yemen has grappled with adopting the WHO/European measures to control this pandemic, resulting in massive devastation to the already weak economy [14]. Yemen declared its first case on 10 April 10 [15]. Up to 25 August 2022, Yemen has registered 11,919 confirmed cases and 2155 deaths due to COVID-19 [16]. However, this seems to be an underestimate, as there is limited COVID-19 testing capacity [17,18].

Individuals react to threatening health information either by surveilling it and respectively taking appropriate measures or by avoidance and denial of such information [19]. Findings from other health disciplines reveal that a noteworthy proportion of the population opts to avoid anxiety-inducing information, such as cancer risk, HIV status, or a genetic disposition to diseases [20,21]. Typically, health information avoidance is an emotionally driven, maladaptive defensive mechanism [19]. According to the information avoidance framework, individuals tend to avoid information when understanding the information is associated with aversive emotions (e.g., acquiring a cancer diagnosis elicits fear) or mandates individuals to take undesired measures [19]. Both responses are highly applicable in the case of COVID-19 in Yemen, as the topic is jeopardizing and instructing individuals to take undesired actions (e.g., social distancing, not going to work). Furthermore, overexposure to health topics that receive a plenitude of attention in the media causes information avoidance [22]. This information avoidance is one of the primary drivers for denying the pandemic in Yemen, especially since areas under the rule of the de-facto authority (DFA) in the north of Yemen (also known as the Houthis) deny the existence of COVID-19 and no testing is taking place [18]. Yemen’s northern governorates, where the Houthis are in power, are home to over 80% of the population–roughly 30 million people [23].

The massive amount of health information in Yemen, which spread during the pandemic on WhatsApp, Facebook, and social gatherings, contained misinformation, disinformation, and rumors, which have directly impacted health (risen morbidity and mortality), caused a misinterpretation of health information & behaviors, distrust in government, science, experts, and public health officers in responses and interventions (vaccine, therapeutics) and a considerable stigma [24]. Certain groups of populations/communities–for example, people with chronic illness/conditions and people with poor digital capacity–are at a higher risk than others of falling for conspiracy theories, false connections, manipulated content, pseudoscience, and other types of infodemic primarily when it’s shared to them from a family member or a dear friend. [12]. These behaviors have seriously jeopardized the efficacy of the government response and public health measures in Yemen and left an increased margin of the population vulnerable and exposed to infodemic, which has induced the Yemeni population not to comply with preventive measures guided by the government, including other health organizations and authorities. Therefore, the here presented study aims to link the impact of the infodemic on people’s incompliance with the COVID-19 preventive measures in Yemen. 

Many previous studies have indicated that low-income countries’ populations did not adhere very long to the European/Chinese model to prevent COVID-19 infection due to socioeconomic factors [25,26]. Still, this is one of the few studies to focus on further factors that might have contributed to the people’s incompliance with preventive measures and disbelief in COVID-19 and its restrictions in Yemen. The presented study aims first to measure the governmental stringency in applying the containment measures, second asses the public compliance with the applied restrictions, and third to have an insight into the dynamic of the COVID infodemic in the context of Yemen and its impact in the public health behaviors and decisions but ultimately to highlight the importance of establishing the basis for infodemic management in Yemen as a mainstream strategy for achieving resilience against misinformation, supporting compliance with governmental public health measures, and building trust in the government and health authorities.

## 2. Materials and Methods

### 2.1. Study Design

This study used a mixed method approach; the quantitative data were taken from multiple sources (The Oxford COVID-19 Government Response Tracker and Google COVID-19 Community Mobility Reports), analyzed, and then complemented by In-depth interviews with Yemeni community and religious leaders as well as social media influencers that was conducted in Yemen from August to December 2021.

### 2.2. Sampling

Purposive sampling was used to select participants who are considered influencers in the Yemeni community to be interviewed for the study. The selection of the participants was based on their influence on the community, reflected by the subscription number on their social media profiles and their religious and community role in Yemen. Sixteen (16) participants were invited to participate first via phone call and then sent an official invitation via Email/WhatsApp.

### 2.3. Data Collection

Quantitative data were first downloaded from the database mentioned in the study design and analyzed in both SPSS and Excel sheets. The in-depth interviews (IDI) were conducted with only twelve (12) out of the sixteen (16) participants who were invited. The other four (4) participants did not participate due to traveling reasons or could not be reached. Initially, the study’s information sheet and consent form was sent to all participants to participate in the interview. After obtaining participants’ consent to participate in this study, eleven (11) interviews were conducted face-to-face in a quiet place without interruption. However, one (1) interview was via Skype. All interviews were audio recorded. Researchers utilized an IDI guide explicitly developed for this study. All the interviews were conducted in Arabic, and the response notes were transcribed in Arabic, then translated into English. Hence, the data was rendered in English for further analysis. The interview audio recordings and notes were securely saved.

### 2.4. Data Analysis and Data Instrument

#### 2.4.1. Stringency Index SI

To assess the intensity of lockdown measures in Yemen, the “Stringency Index “of the strictness of containment measures has been calculated by The Oxford COVID-19 Government Response Tracker [27] during the study period from the nine subsequent metric indicators: Closure of schools, workplaces, public events, and public transportation; Restrictions on gatherings, internal movements, and international travels; Public information Campaigns; and the quarantine requirements. Calculating the index followed the methodology described by Hale et al., which evaluated the intensity of governmental measures on a scale from 1 to 100, with 100 demonstrating the maximum application of all indicators mentioned overhead [28]. In addition, Yemen’s governmental response data were collected from The Oxford COVID-19 Government Response Tracker [27] into an excel sheet, and a curve was obtained. However–other health control measures, such as masks/hygiene, are not included in the SI index.

#### 2.4.2. Google COVID-19 Community Mobility Reports

After the Yemeni government installed containment measures, peoples’ compliance with the lockdown was documented by aggregated data from the Google COVID-19 Community Mobility Reports to measure the change in their mobility [29]. In this report, the percentage of shifts in visiting various establishments and places (i.e., retail and recreation, grocery and pharmacy, parks, transit stations, workplaces, and residential areas) were compared to the baseline level and were estimated by aggregating the location data of Google account users. These data were collected from Google mobility Reports into SPSS, where Yemen-specific data were extracted into an Excel sheet where the curves were obtained.

#### 2.4.3. Offline Social Listening “In-Depth Interviews”

Social listening is identifying, assessing, listening to, and working with communities to understand and respond to concerns that have been paramount during a health crisis. [30].

There are two types of social listening:

○ Online social listening, which includes (tracking users’ reactions to relevant COVIDs topics on social media platforms augmented by artificial intelligence; monitoring applications) [31].
○ Offline social listening, which includes (in-depth interviews, surveys, and audience observation…).

In this study, we focus on offline social listening to get an insight into the populations’ behavioral change during the pandemic. Twelve in-depth interviews were conducted with Yemeni influencers: two Community leaders, two religious leaders, one school principal, two social activists, and five of the most popular social media influencers in Yemen.

A research team member transcribed all twelve (12) interviews verbatim. For anonymity, each interviewee was given a unique secure number (from one to twelve).

The twelve interviews were first transcript and translated into English. Then, four data analysis steps were followed to gain insight into the participants’ perceptions of the impact of COVIDs’ infodemic in Yemen:

○ Interpreting the data by reading each transcript and underlining statements using NVIVO.
○ All underlined statements were coded across each interview, undergoing inductive analysis.
○ All codes were grouped into six themes.
○ All statements in all themes were read to reflect the overarching participant’s perceptions about the impact of the COVIDs infodemic in Yemen.

## 3. Results

### 3.1. The Containment Measures in Yemen

**Figure 1:**
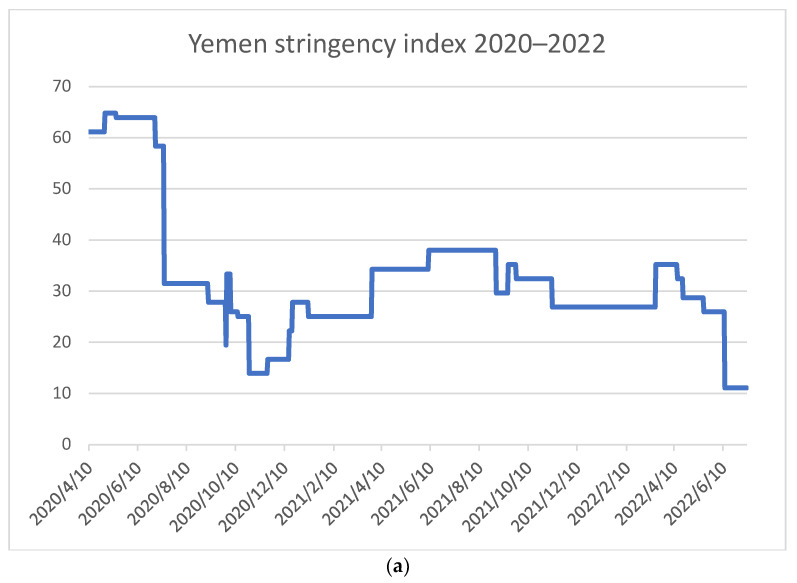

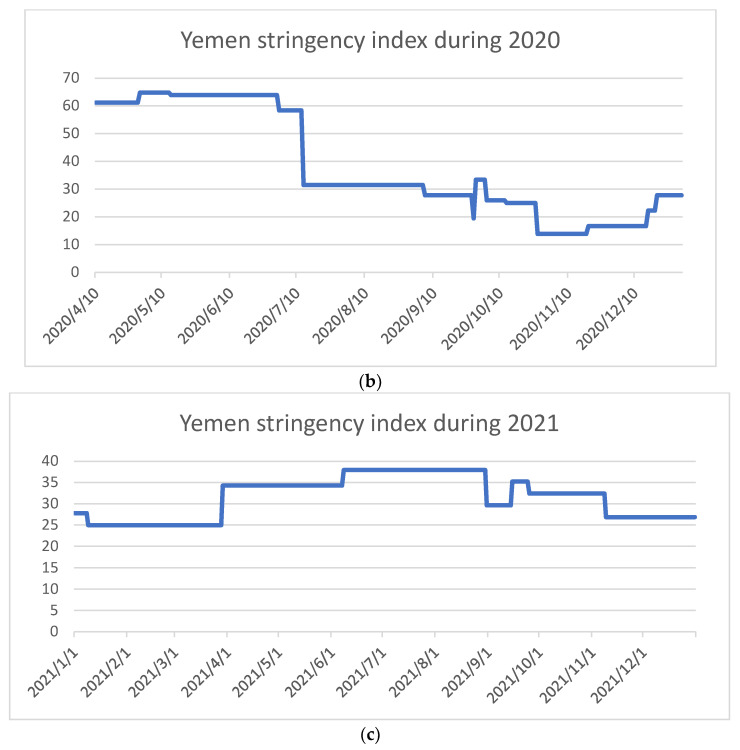
(**a**) Yemen Stringency Index; (**b**) Yemen Stringency Index from 10 April 2020 till the end of 2020; (**c**) Yemen Stringency Index during 2021.

The stringency Index Figure 1 shows the containment measures in Yemen over time (April 2020–June 2022). The government started applying preventive measures with a relatively high index of 60–65 in early April 2020 as the pandemic had just begun and the government declared its first case. Though three months later, containment measures were dropped to an index of 30s in July 2020 and continued to decrease gradually until an index of 10–20 was reached in October 2020. From 2021 till mid-2022, the SI fluctuated between 25 s–35 s until it dropped and reached an index of almost ten by the middle of 2022. 

### 3.2. The Human Movement after the Introduction of Containment Measures during 2020 and 2021 in Yemen

**Figure 2:**
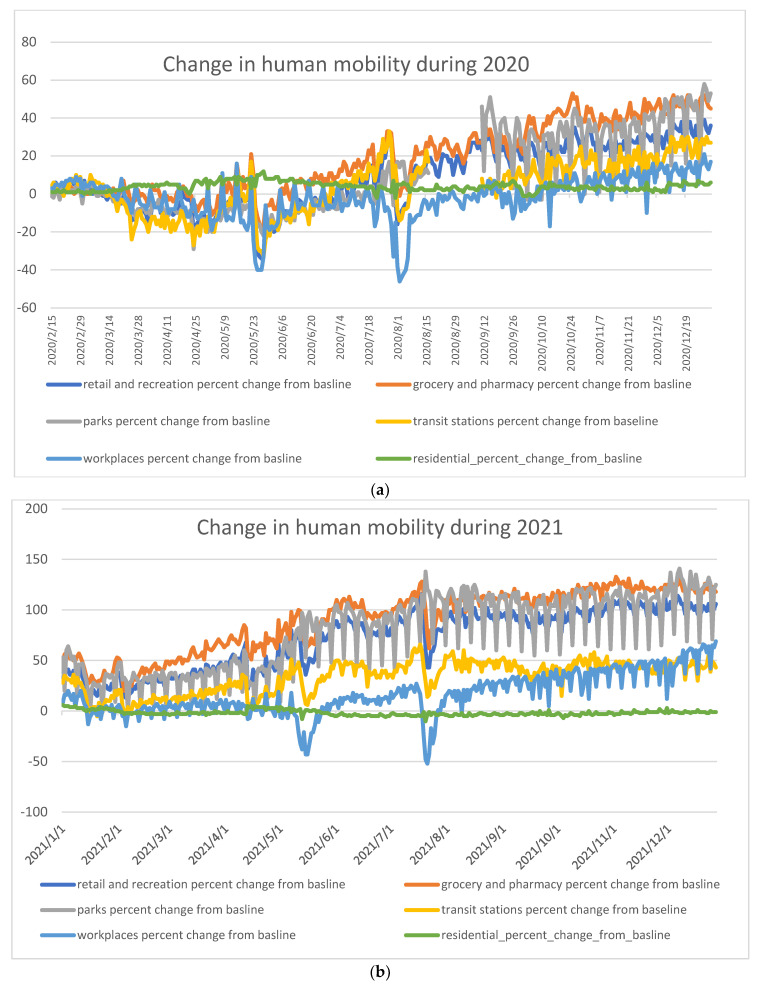
(**a**) Human mobility after the start of containment measures in Yemen during 2020. Blue = Retail and recreation, Red = Grocery and pharmacy, Gray = Parks, Orange = Transit stations, Light blue = Workplaces, Green = Residential; (**b**) Human mobility after the start of containment measures in Yemen during 2021. Blue = Retail and recreation, Red = Grocery and pharmacy, Gray = Parks, Orange = Transit stations, Light blue = Workplaces, and Green = Residential.

The Human mobility (Figure 2a,b) show the change in the movement of the population in comparison to previous years. Looking into (Figure 1a and Figure 2a together), it is to be noticed that even during March, before the announcement of COVID exitance in Yemen, the population movement decreased slightly more than usual. 

From 10 April till the end of 2020 (specifically April–July), when the SI was at its highest, the containment policy led people to substantial compliance with movement restrictions (Figure 1a and Figure 2a). People tended to stay at home and followed many extra-domestic activities with limited recreational activities; as requested by the government, people reduced going to work, using pharmacies/groceries, and used public transport less than usual except during the two Eid holidays in May and July when transit and shopping in groceries peaked (Figure 1a and Figure 2a). 

During the second half of 2020, the government eased the preventive measures into an index of 30 s, then as low as 13. Consequently, the population compliance with movement restrictions curve slowly peaked gradually as their movement began to increase. As a result, they stayed less at home and gradually went back to work, transit, and other recreational activities (Figure 1a and Figure 2a). By the end of 2020, the government raised containment measures to an index of 28, but poor compliance is to be noticed by the public as their movement continued to increase (Figure 1a and Figure 2a).

Accordingly, (Figure 1b and Figure 2b) show a continuous substantial increase in the movement pattern as people in 2021 showed much less compliance despite the fact that the government continued to implement more restrictions to an index of 38.

## 4. Qualitative Themes

### 4.1. People’s Belief in COVID-19 in Yemen

#### 4.1.1. COVID-19 Will Not Reach Yemen

The people’s belief in COVID-19 (Table 1, Section 1) passed through different stages in Yemen; at the beginning of the Global pandemic in early 2020, people were sure that COVID-19 would not reach Yemen due to the ongoing siege on the borders.

*“…I have made a YouTube video interviewing people, where I asked them: what do you think of COVID? do you have any concerns about COVID? or are you afraid of COVID? Their answers were as follow: we have no fear of COVID, we didn’t have any fear from the air jets and their air strikes and bombs falling down on us and so a disease in China will not scare us, it is impossible for the disease to reach Yemen…”*

informant 5

#### 4.1.2. People Believed in COVID after the Official Announcement of the First Case 

However, the previous belief changed immediately after the announcement of the first case in Yemen. Some people began to understand the real situation, and others still didn’t believe in COVID-19 until having a close family member hospitalized due to COVID-19. Nonetheless, the majority kept denying COVID-19.

*“…after the ER in hospitals were full with COVID patients and there were many deaths, some started to understand the situation but some just said this is a normal disease like any other disease and if we were meant to die then its all up to Allah…”*

informant 3

*“…people started to believe in COVID-19 when some of their relatives got the disease. Only then they have realized that it actually exists…”*

informant 8

### 4.2. Compliance with Preventive Measures

#### 4.2.1. Partial Compliance during the First Wave

Although the majority of the population didn’t believe in COVID-19, there were times when they complied (Table 1, Section 2) because the government had placed some restrictions and preventive measures but also out of fear after seeing an increasing rate of deaths amongst their narrow community circles.

*“…Honestly there were some sort of compliance with COVID preventive measures. But only a small fraction of the population complied with those restrictions, and from this fraction some complied very strictly and some has partially complied…”*

informant 1

*“…This compliance was during the 1st wave from April till August in which many old people died, and people were denying COVID but deep inside they were afraid to die, so there was fear of dying of COVID which caused them to also deny COVID, because people deny what they are afraid of…”*

informant 6

#### 4.2.2. The Preventive Measures Applied by the Government Intensified the Public’s Compliance

The main driver of people’s compliance was the government’s strictness in applying preventive measures (Table 1, Section 2). People’s compliance with preventive measures was only witnessed during the first wave in Yemen with the government’s measures to control the outbreak. For instance, schools, universities, and mosques were closed, and masks and sanitizers were mandatory at certain places. As a result, compliance with social distancing was practiced partially; people started to avoid crowded places, many people worked from home, the number of people using public transport decreased, and many people canceled their wedding ceremonies. However, only a tiny fraction of the population remained home, and this compliance didn’t last long when the government eased those restrictions three months later.

*“…people were waiting for the government to tell them that COVID does really exist even if it’s there and they can see it, they want the government to start announcing its existence…”*

informant 5

*“…People complied very strictly with preventive measure at the beginning, it was not allowed to go to markets without masks and sanitizers, but now all these precautions have vanished completely…”*

informant 3

*“…it was a compliance to only follow the rules and not to avoid COVID, for example children were not allowed to go to schools but they would go out and play at the street with other children…”*

informant 10

*“…many people were planning to celebrate their weddings in huge weeding halls but cancelled it and only made a small ceremony for close friends and family…”*

informant 6

#### 4.2.3. Less Compliance during the Second Wave 

Almost all the previous social compliances with preventive measures witnessed during the first wave in 2020 disappeared during the second wave in 2021(Table 1, Section 2). However, it is assumed that more people still got sick during the second wave, as testified by the interviewees.

*“…when the second wave came no one cared at all, even at the gate of the university the security would ask you where’s your mask and I would show them the mask in my hand without out wearing it and they would let me in…”*

informant 1

*“…I even had COVID for a whole month I had all the COVID symptoms, and all my nine colleagues got sick, and so during the second wave a huge proportion of the population got COVID because no one cared for preventive measures and restrictions in the universities, markets or mosques…”*

informant 1

*“…my whole family got sick all my colleagues got sick almost half of my cohort in the university got sick no one who I have known didn’t get sick at that time during the 2nd wave…”*

informant 2

#### 4.2.4. Government Denial of COVID-19 and Limited Capacity to Inform the Public of Recent Updates

The de facto state in the north has eased some precautions and denied that there is COVID in Yemen (Table 1, Section 2). The situation in the south was not different because there were only a few testing facilities, so only a few cases were to be announced. All these combined factors gradually caused people’s fear and compliance to vanish when the government applied precautions again in 2021; during the second wave, the public did not respond.

*“…I think that people showed more compliance during the 1st wave because schools and universities were forced to shut down by the government, but during the second wave, those restriction were dropped off and eased…”*

informant 2

*“…many got sick, and unfortunately, no one was declaring the number of cases or deaths although many died during that period but no one knew. If they were to announce the actual cases and deaths people would have paid attention to COVID measures. At that time everyone knew how badly affected our health system was due to the ongoing war and the lack of medical supplies and people were afraid of going to hospitals…”*

informant 10

### 4.3. The Impact of Infodemic on Compliance

#### 4.3.1. Lack of Access to Reliable Information and Information Voids

There was a tremendous lack of access to reliable sources from the beginning of the pandemic (Table 1, Section 3). The population did not know where to find answers to their questions and concerns. Moreover, many of the population still need to gain the knowledge to find accurate information from reliable sources, navigate information on social media, and the ability to fact-check information on social media, especially during emergencies when social media is full of misinformation. What made the situation worse was the fact that People wanted to be the first to publish information, regardless of the credibility and quality of information they had.

*“…unfortunately, during COVID our government and health ministry didn’t specify some sources or websites in which a person could search in for reliable information or guidelines…”*

informant 2

*“…our community is still let me put it this way, we are still lacking the means and tools that help us to reach accurate information and so the search capability for our community is still very poor and the great majority are relying on social media and that’s a huge mistake, but there are only few who are specialized or aware enough to look for the information from its main source…”*

informant 4

*“…unfortunately in our community even Journalists and media creators don’t check their sources or they even publish without any sources, those behaviours have contributed to the spread of false information, many popular influencers, journalists, social activists or even doctors have spread false information with no sources and people trusted their misinformation…”*

informant 12

#### 4.3.2. The Pandemic’s Indicator in Yemen and How the Public Got Their COVID Updates

The fact that the government was not fairly transparent in sharing updates or official reports of cases or deaths has forced people to rely more heavily on social media to be aware of the situation (Table 1, Section 3). Whether there were more or fewer funerals than usual announced on Facebook was the leading indicator for Yemeni people to measure the situation. Yemeni doctors would also unofficially report on social media how full hospitals were with COVID-19.

*“…the governmental official media channels tried to cover up any information about COVID-19…”*

informant 8

*“…Unfortunately I am from those people that I measure the extent to which COVID-19 is spread from the number of deaths and funerals posted on Facebook, because there were no reliable source in which I can use to give me true daily updates of the cases and deaths or recovery numbers like other countries…”*

informant 5

*“…doctors in Yemen has done something to aware people in stating that hospitals were full with COVID-19 cases, that information were published only on social media…”*

informant 9

#### 4.3.3. Traditional Measures Are Prioritized over Preventive Measures

People tend to substitute proper guidelines and preventive measures with misleading guidelines and rumors from social media like herbal remedies, drinking water, vinegar, and eating onion, garlic, and other herbs instead of following the guidelines suggested by the government or the WHO (Table 1, Section 3).

*“…when information circulate the internet that drinking water could help you avoid COVID people will tend to drink more water rather than complying with hard and strict measures like wearing a mask all day long. So, in short people take new easy rumours like herbal remedies and traditional methods as a substitute to the preventive measures advised by the WHO…”*

informant 7

*“…one of the main reasons that stopped people from complying with preventive measures is the overwhelming amount of fake news on social media…”*

informant 2

#### 4.3.4. COVID-19 Misinformation on Social Media Changed the Public Perception of COVID-19

The amount of misinformation during the first wave has caused people to overestimate COVID-19′s fatality and transmission rate (Table 1, Section 3). Still, after seeing that of all those who got sick many had only mild or asymptomatic COVID-19, this has caused people to deny the seriousness of COVID again and to think of it as an illness similar to the regular flu. COVID was also called by different names like Gasea’a, which translates into the English word (passing/going away) to make it sounds light and it will go away.

*“…After many people had COVID-19 and got cured many started to believe that COVID is like a normal flue or a cold…”*

informant 11

*“…our community came up with new names for COVID like Mokarfas or Gasea’a which is supposed to be a lighter version of a new disease but it’s not the COVID everyone is afraid of…”*

informant 1

#### 4.3.5. Social Pressure and Stigma against Anyone Who Might Comply with the WHO Guidelines

Moreover, the social pressure and the stigma on those who want to comply with preventive measures have increased dramatically (Table 1, Section 3). The dominant Practice and belief were that if you comply with preventive measures, people would suspect that you have COVID-19 and will start to avoid you, and you will be bullied and accused of causing fear.

*“…the rule was if you wear a mask then you have COVID…”*

informant 2

*“…people bullied me and accused me of having COVID and I thought by complying with COVID measures I will be a source or stress to people around me and took the mask off…”*

informant 7

*“…. In short even if you want to act responsibly and you want to comply with preventive measure there will be a huge social pressure on you not to comply…”*

informant 11

*“…many wanted to wear mask but out of shame and fear to be bullied they stopped complying with wearing masks in public areas…”*

informant 9

## 5. Discussion

Through listening to our targeted audience and identifying individuals’ information gaps, needs, and behaviors together with our quantitative results (Stringency Index and Human movement), the early community movement reduction during the period of 29 February–10 April 2020, before the application of preventive measures by the government, reflects a high level of awareness and anticipation of the general public of the COVID-19. 

From the 10 April 2020, more people complied as the government restricted their movement. The population’s movement in Yemen increased right after the government eased restrictions three months after the announcement of the first local case, which is to be expected as the population followed the government rules. Another study analyzed data from Four HICs (Germany, Sweden, Italy, and South Korea) and five LMICs (Mexico, Colombia, India, Nigeria, and Nepal) [32] and showed that in all countries, people complied with restrictions on movement applied by governments. In Yemen, the people’s movement continued to increase, though the government retained some restrictions on movement from April 2021 till April 2022; moreover, our qualitative data showed no compliance with wearing masks or practicing hygienic and other preventive measures. 

Evidence from the literature shows that the beginning of a health crisis is a critical period when transparent use and communication of evidence and insights into decision-making processes can affect perceptions of the government’s pandemic response and high transparency levels in their communication to encourage public precautionary cooperation [33,34]. Unfortunately, this was not the case in Yemen, where little effort was put into spreading awareness. For example, although the Yemeni Supreme National Emergency Committee for Covid-19 had an official account on Twitter and a WhatsApp group, where they routinely share cases and death reports [35], none of our informants were aware of its existence or have seen their reports. This reflects that those reports were also poorly promoted and not widely published; in addition, Twitter is the less used social media platform in Yemen. A study conducted in Ethiopia amongst the university community to measure the level of compliance with COVID preventive measures has indicated that working on the community’s knowledge, practices, and attitude about COVID preventive measures through media campaigns ultimately increases compliance [36]. However, the witnessed incompliance and reduction in following the rules in Yemen also reflect a relative disbelieve in COVID-19 and mistrust in health authorities and guidelines.

A study conducted during the beginning of the COVID pandemic ascertained that the public’s collective attention to the pandemic typically heightened immediately after the announcement of the first local case [37]. The same study also found that the rising level of information-seeking dramatically declines soon after, even though many announcements of school closure or other mitigation strategies follow. This indicates that increased attention is only short-lived and acquires a rapid response to any misinformation [37,38], as this behavior of the public seeking for information was not backed up by the status of COVID-19 (example–the number tested, number infected, number hospitalized, number deaths, etc.) of the country from the authority as many countries were trying to inform their citizens of the actual situation. Although the mentioned study was conducted in the US, an entirely different setting and culture than in Yemen, the social behavior of the population in Yemen proved to be similar during health crises as the population in Yemen lost interest in showing compliance or following the pandemic news very early.

A study conducted in brazil showed that the significant underreporting rates made it impossible to implement a more effective pandemic response [39]. Likewise, the authorities in Yemen had contributed indirectly to the spread of misinformation and ultimately to the denial of COVID-19 by underreporting cases and mortality due to low testing facilities/supplies or political reasons, which hindered a more effective response. Another study also suggests that health officials are urged to be transparent about the situation and work away to solve panic by providing accurate information sources people should return to whenever the situation looks dubious [40].

A study that focused on the impact of rumors and misinformation on social media yielded that the COVID-19 pandemic has not only caused significant challenges for health systems all over the globe but also fuelled the surge of countless rumors, hoaxes, and misinformation regarding the etiology, outcomes, prevention, and cure of the disease [41]. Unfortunately, this outbreak of misinformation is masking healthy behaviors and promoting erroneous practices that expand the spread of the virus and ultimately result in poor physical and mental health outcomes among individuals [41]. In Yemen, many rumors, satires, fabricated content, false connections, propaganda, conspiracy theories, and other types of infodemic caused the people to look for other measures to prevent themselves from COVID, like herbal remedies or other traditional practices that were circulating the social media and extensively discussed during social gatherings.

Although studies from the literature indicate the impact of social pressure and stigma during COVID-19, fewer showed enormous social pressure on those who thought to take precautions or tried to get informed about COVID [42,43,44]. This was the case in Yemen, where a person would be accused of causing stress, fear, or foreign propaganda for showing any compliance. In our opinion, the reason why the most vulnerable strata of the population tend to follow certain behaviors during health crises (e.g., denying COVID-19, stigmatizing others for following the WHO guidelines, spreading misinformation, and promoting rumors) is that they are afraid of lockdown measures, which shall worsen their economic situation and hinder their daily life. In Yemen, although preventive measures were eased, misinformation and social pressure continued out of fear of a second or a third wave that might bring back more restrictions. Therefore, a behavioral change model and human-centered interventions that put poverty and economic factors into account are needed to tackle the infodemic in Yemen.

The exposure to misinformation daily during the struggle to find information about COVID in Yemen has caused some people to be highly stressed, which is also to be witnessed in the literature as an early study in the pandemic showed that subsyndromal mental health problems are a typical response to the COVID-19 pandemic and when planning for a governmental response the needs of the concerned vulnerable people and the required preventive guidelines must be taken into account [45].

In Yemen, from the announcement of the first case, the authorities dealt with the situation as if it was only one case isolated in a hotel, and there should be no fear of a further spread. A few months later, the De-facto authorities in the north had denied that COVID-19 exists, and only the southern governorates were reporting COVID cases and deaths. However, these reports were made for authorities and international organizations rather than for the public. Therefore, the majority of the public hasn’t received COVID updates on a regular basis. Consequently, people didn’t realize the magnitude of the problem, and the majority of the population didn’t believe in COVID completely.

With the understanding of the detrimental effects the infodemic could have on the population, their health-seeking behavior, and their health status, WHO (2021) provided guidelines on how the country/authority may address this important problem. This guideline has five streams to be adapted based on the country/area’s situation. The response/efforts/intervention made by the Yemeni authority seems to be inadequate. The capacity building of the government to manage upcoming infodemics and develop resilience against misinformation need advanced preparedness. The WHO suggests that taxonomies and classifications are to be standardized and new metrics are to be developed to quantify the infodemic at the early stages of any health crisis with the focus that data should even be quantified from multiple sources for analysis [46]. However, in conflict settings like Yemen, where the government is underreporting and not broadcasting routine reports in a broader context, e.g., per SMS, Newspapers, or weekly media conference, the previously mentioned infodemic control guidelines might not be sufficient to control misinformation when people still fail to acknowledge that there is an outbreak.

Combating the Infodemic demands robust efforts and substantial cooperation in Yemen; as misinformation surpasses all households in Yemen, so shall the actions and activities that will blunt its damaging impacts. In collaboration with those who occupy the most elevated seats of power to the individuals who bear the trust of the communities where they live, infodemic management has a role as part of a comprehensive emergency response approach.

Although our quantitative data comes from reputable sources, which were also used in many other studies in the literature, we believe that the Google community movement tool only captures smartphone users, which means the most vulnerable strata of our community, who have no access to technology might not be represented in our graphs. Therefore, in-depth interviews with religious and community leaders from those communities were incorporated to address the issue of their compliance with preventive measures on movement. However, social media influencers were also key to sharing their insight into what health information, including misinformation, is being shared and circulating on social media and instant messaging to measure their impact on people’s behavior during COVID. As the case with in-depth interviews, which are retrospective, our qualitative results might be disclosed to recall bias. Nevertheless, the interviewers were trained in interview skills before conducting this study, and each interview was followed by peer debriefing.

## 6. Conclusions

In conclusion, although Yemen’s fragile health system, political conflict, humanitarian crises, and economic situation have contributed to the denial of COVID-19 and were the initial drivers not to prioritize COVID-19 during the first days of the pandemic [44], the population’s information-seeking behaviors, the government’s low transparency, and how people filled this vacuum (e.g., looking for any evidence to support their state of denial) has intensified the infodemic and its consequence in Yemen. In conflict settings, the Chinese/European model (lockdown) to mitigate COVID-19 is not applicable [24], and the most vulnerable strata of the population, who represent the majority, are always afraid to be left behind, which is encouraging them to promote any misinformation that may bring life back to normal and end their fear. This led the COVID infodemic in Yemen to be multi-facet and impact individuals and communities, causing: Physical harm (e.g., limited, accurate knowledge about available treatments, misplaced actions, and dismissal of proven public health measures.), social harm (e.g., victimization, stigma, and violent aggression among community members.), political harm (e.g., limited trust in officials, rejection of official guidelines, disregard of government-led responses.), and psychological harm (e.g., mental health disorders due to extreme anxiety and panic.) Therefore, Yemen is in critical need of establishing a dynamic infodemic response team, which shall continuously review and adapt related guidelines, synthesizing existing evidence and guidance for the Yemen-specific contexts, especially as it relates to individuals and vulnerable communities, promoting a culture of transparency and continuous monitoring of misinformation online/offline, informing the government of any existing information voids, testing possible ways to address infodemics including capacity building for raising the population immunity against misinformation.

## Figures and Tables

**Table 1:** Summaries the themes of the qualitative in-depth interviews.

Qualitative Themes	Specific Idea/Code
People’s Belief in COVID-19 in Yemen.	COVID-19 will not reach Yemen.
	People believed in COVID after the official announcement of the first case.
2. Compliance with preventive measures.	Partial compliance during the first wave.
	The Preventive measures applied by the government intensified the public’s compliance.
	Less compliance during the second wave.
	Government denial of COVID and limited capacity to inform the public of recent updates.
3. The impact of infodemic on compliance.	Lack of access to reliable information and information voids.
	The pandemic’s indicator in Yemen and how the public got their COVID updates.
	Traditional measures are prioritized over preventive measures.
	COVID-19 misinformation on social media changed the public perception of COVID-19.
	Social pressure and stigma against anyone who might comply and follow the WHO guidelines.

## Data Availability

The data that support the findings of this study are not publicly available due to privacy or ethical restrictions.
